# Response Surface Methodology for Optimizing the Production of Biosurfactant by *Candida tropicalis* on Industrial Waste Substrates

**DOI:** 10.3389/fmicb.2017.00157

**Published:** 2017-02-07

**Authors:** Darne G. Almeida, Rita de Cássia F. Soares da Silva, Juliana M. Luna, Raquel D. Rufino, Valdemir A. Santos, Leonie A. Sarubbo

**Affiliations:** ^1^Northeast Biotechnology Network, Federal Rural University of PernambucoRecife, Brazil; ^2^Advanced Institute of Technology and InnovationRecife, Brazil; ^3^Center of Sciences and Technology, Catholic University of PernambucoRecife, Brazil

**Keywords:** biosurfactant, *Candida tropicalis*, optimization, scale up, oil dispersion

## Abstract

Biosurfactant production optimization by *Candida tropicalis* UCP0996 was studied combining central composite rotational design (CCRD) and response surface methodology (RSM). The factors selected for optimization of the culture conditions were sugarcane molasses, corn steep liquor, waste frying oil concentrations and inoculum size. The response variables were surface tension and biosurfactant yield. All factors studied were important within the ranges investigated. The two empirical forecast models developed through RSM were found to be adequate for describing biosurfactant production with regard to surface tension (*R*^2^ = 0.99833) and biosurfactant yield (*R*^2^ = 0.98927) and a very strong, negative, linear correlation was found between the two response variables studied (*r* = −0.95). The maximum reduction in surface tension and the highest biosurfactant yield were 29.98 mNm^−1^ and 4.19 gL^−1^, respectively, which were simultaneously obtained under the optimum conditions of 2.5% waste frying oil, 2.5%, corn steep liquor, 2.5% molasses, and 2% inoculum size. To validate the efficiency of the statistically optimized variables, biosurfactant production was also carried out in 2 and 50 L bioreactors, with yields of 5.87 and 7.36 gL^−1^, respectively. Finally, the biosurfactant was applied in motor oil dispersion, reaching up to 75% dispersion. Results demonstrated that the CCRD was suitable for identifying the optimum production conditions and that the new biosurfactant is a promising dispersant for application in the oil industry.

## Introduction

Surfactants are amphipathic molecules that reduce the surface and interfacial tensions of liquids (Santos et al., 2013; Silva et al., 2014a). Such compounds have a predilection for interfaces of dissimilar polarities (liquid–air or liquid–liquid) and are soluble in both organic (non-polar) and aqueous (polar) solvents (Luna et al., 2013). Due to these properties, surfactants have a wide variety of applications in medicine, household products, agriculture, food products, cosmetics, pharmaceuticals, and the petroleum industry (Rufino et al., 2014).

Biosurfactants are surfactants of biological origin. Microorganisms (bacteria, yeasts and fungi) are known to produce biosurfactants (Luna et al., 2013), which are classified as glycolipids, lipopeptides, fatty acids, polymers, or particulate compounds (Rufino et al., 2014). Although the majority of biosurfactants have been reported in bacteria, the pathogenic nature of some producers restricts the wide application of these compounds (Toribio et al., 2010; Sharma et al., 2016). Given the industrial importance of yeasts and their potential to biosurfactant production, a growing number of aspects related to the production of biosurfactants from yeasts have been the topic of research during the last decade (Amaral et al., 2010). Some species of *Candida*, such as *Candida bombicola* (Roelants et al., 2013; Luna et al., 2016), *Candida glabrata* (Luna et al., 2009; Gusmão et al., 2010), *Candida lipolytica* (Santos et al., 2013; Rufino et al., 2014), *Candida sphaerica* (Sobrinho et al., 2013a; Luna et al., 2015), *Candida utilis* (Campos et al., 2013), *Candida guilliermondii* (Sitohy et al., 2010), *Candida antarctica* (Kim et al., 2002; Hua et al., 2003), and *Candida tropicalis* (Batista et al., 2010; Priji et al., 2013) are known to produce biosurfactant.

Biosurfactants offer a number of advantages over chemical surfactants, such as better biodegradability, environmental compatibility, low toxicity and highly specific activity under extreme conditions of temperature, pH and salinity (Banat et al., 2010). Despite the advantages, biosurfactants are not yet competitive with their synthetic counterparts due to the high production costs. Therefore, most commercially available surfactants are synthesized from the petrochemical industry, which currently accounts for 70–75% of all surfactants used in industrialized nations (Campos et al., 2013). However, with the increased awareness among consumers for environmentally friendly compounds, industries are presently seeking to replace some or all chemical surfactants with sustainable biosurfactants (Marchant and Banat, 2012; Sekhon et al., 2012). As a result, the global market for biosurfactants has been growing in recent years. For instance, in 2011, the worldwide biosurfactant market was worth approximately US$ 1.7 billion, which is expected to reach US$ 2.2 billion by the year 2018 (Sekhon et al., 2012).

The development of economical processes for biosurfactant production has become the key factor to reducing costs and increasing competitiveness. Industrial wastes have attracted considerable interest from researchers as low-cost substrates for this purpose, as the substrate generally accounts for up to 50% of the final production cost (Das et al., 2009; Rufino et al., 2014). Residues, such as corn steep liquor (Silva et al., 2013), glycerol (Silva et al., 2010), clarified cashew apple juice (Oliveira and Garcia-Cruz, 2013), vinasse (Oliveira et al., 2013), cassava wastewater (Barros et al., 2008), soybean oil refinery residue (Luna et al., 2011a), ground-nut oil refinery residue (Sobrinho et al., 2008), animal fat (Santos et al., 2013), vegetable fat (Gusmão et al., 2010), waste frying oil (Batista et al., 2010), and molasses (Santos et al., 2010) have demonstrated excellent results when used for biosurfactant production by microorganisms.

Response surface methodology (RSM) has been effectively employed to reduce the production cost of biosurfactants through the selection of balanced proportions of the constituents of the culture medium and the optimization of culture conditions (Najafi et al., 2011; Kim and Kim, 2013; Silva et al., 2013; Kumar et al., 2015). RSM constitutes a collection of statistical techniques for designing experiments, building models, simultaneously evaluating the effects of factors and establishing optimum conditions. A central composite rotational design (CCRD) is used with RSM to examine the relationship between one or more response variables and set of quantitative experimental factors (Kim and Kim, 2013). As the correlation between the response and independent variables is generally unknown at the onset of a process, the first step in RSM is to approximate the function (response) by analyzing the factors (independent variables) (Najafi et al., 2010; Silva et al., 2013).

Large-scale biosurfactant production seems to be an effective strategy to overcome the competitiveness with their synthetic counterparts. There is a lack of fundamental knowledge about biosurfactant production scaling up. In order to develop suitable technology for possible commercialization, it is essential to carry out tests in bioreactors that are systems which allow a larger control of parameters affecting rates of microbial growth. The use of bioreactors becomes an alternative even more attractive and promising when associated to previous optimization of the culture condition using shake flasks to reduce the cost and process time. Then the scale up in bioreactors will lead to an operational facilitation in the implementation of the industrial-scale production (Mukherjee et al., 2006; Amani et al., 2010; Chikere et al., 2012; Luna et al., 2015).

The aim of the present study was to optimize biosurfactant production by the yeast *C. tropicalis* UCP0996 in flask experiments using a combination of CCRD and RSM and test the best response in large scale conditions. The biosurfactant ability as an oil dispersant was also evaluated.

## Materials and methods

### Materials

All chemicals were reagent grade. Growth media were purchased from Difco Laboratories (USA). Canola waste frying oil was obtained from a local restaurant in the city of Recife, state of Pernambuco, Brazil, stored according to the supplier's recommendations and used without any further processing. Corn steep liquor was obtained from Corn Products do Brasil in the municipality of Cabo de Santo Agostinho, state of Pernambuco, Brazil. Sugarcane molasses was obtained from a local sugar mill in the municipality of Vitória de Santo Antão, state of Pernambuco, Brazil. Corn steep liquor is 21–45% protein, 20–26% lactic acid, 8% ash (containing Ca^2+^, Mg^2+^, K^+^), 3% sugar and has low fat content (0.9–1.2%), while sugarcane molasses is 75% dry matter, 9–12% non-sugar organic matter, 2.5% protein, 1.5–5.0% potassium and 1% magnesium, phosphorus, and calcium (Santos et al., 2016). Canola waste frying oil is composed by the following fatty acids: 53–70% oleic acid, 15–36% linoleic acid, and, 5–13% linolenic acid. Seawater was collected near the Suape Port, located in the municipality of Cabo de Santo Agostinho, in Pernambuco state, Brazil. Water samples were collected and stored in plastic bottles of 5 L.

### Yeast strain and preparation of inoculum

A strain of *C. tropicalis* UCP0996 was provided from the culture collection of the Catholic University of Pernambuco, Recife city, Pernambuco, Brazil. The microorganism was maintained at 5°C on yeast mold agar (YMA) slants containing (w/v) yeast extract (0.3%), malt extract (0.3%), tryptone (0.5%), D-glucose (1.0%), and agar (5.0%). Transfers were made to fresh agar slants each month to maintain viability. Inoculum was prepared by transferring cells grown on a slant to 250-ml Erlenmeyer flasks containing 50 ml of yeast mold broth (YMB). The initial pH of YMA and YMB media was adjusted to 5.5. The cultivation conditions for the seed culture were 28°C, 200 rpm and 24 h of incubation.

### Production medium

The composition of the production medium varied according to the experimental design described below. Sugarcane molasses, corn steep liquor and canola waste frying oil were dissolved in distilled water and the pH was adjusted to 5.5 by the addition of 1 M NaOH solution or 1 M HCl solution. The medium was sterilized by autoclaving at 121°C for 20 min. Aliquots of the YMB suspension containing 10^8^ cells/mL of *C. tropicalis* UCP0996 (varying in accordance with the experimental design) were used to inoculate 500-mL Erlenmeyer flasks containing 100-mL of production medium. The surface tension of the production medium before oil addition and inoculation was 55 mNm^−1^. Cultivation was carried out at 28°C with agitation at 200 rpm for 120 h in a Marconi MA832 shaker (Marconi LTDA, Brazil). No adjustment of pH was performed during cultivation. At the end of fermentation, samples were taken from the liquid culture to determine the surface tension and biosurfactant yield, as described below.

### Optimization of biosurfactant production using RSM

A CCRD was used to determine the effects and interactions of four factors for biosurfactant production. Sugarcane molasses, corn steep liquor and waste frying oil concentrations and inoculum size were the independent variables. Surface tension and biosurfactant yield were the response variables. In this design, a set of 30 experiments was performed, with four replicates at the central points. The statistical analysis of the four replicates gives an indication of the experimental error of the production technique. The range and levels of the components (factors or independent variables) are given in Table 1. Each factor in the design was studied on five levels (−2.0,−1.0, 0,+1, and +2), with zero as the central coded value. These levels were based on results obtained in preliminary experiments. The optimum values from the CCRD were obtained by solving the regression equation and analyzing the response surface contour plots. Analysis of variance (ANOVA) with 95% confidence intervals was used to determine the significance of the effects. ANOVA, the determination of regression coefficients and the construction of graphs were performed with the aid of the Statistica® program, version 12.0.

**Table 1:** **Experimental ranges and levels of independent variables for central composite rotational design used in optimization of biosurfactant production by *C. tropicalis* UCP 0996**.

**Variables**	**Range and levels**				
	**−2**	**−1**	**0**	**+1**	**+2**
Sugarcane molasses (%), *X_1_*	2	2.5	3	3.5	4
Corn steep liquor (%), *X_2_*	2	2.5	3	3.5	4
Waste frying oil (%), *X_3_*	2	2.5	3	3.5	4
Inoculum size (%), *X_4_*	1	2	3	4	5

### Determination of surface tension

Surface tension was determined in the cell-free broth obtained by centrifuging the cultures at 5000 × g for 20 min. Surface tension was determined with a Tensiometer (Sigma 700, KSV Instruments Ltd., Finland), using the Du Nouy ring method at room temperature.

### Scale up of biosurfactant production

Scale up of biosurfactant production was carried out in both 2.0 L bioreactor TEC-BIO (TECNAL, Brazil) and 50 L bioreactor MA 502/50 L (Marconi LTDA, Brazil), containing 1.0 and 25 L production medium, respectively. The substrates used as carbon and nitrogen sources, as well as the inoculum size employed were selected from results obtained by the previous experimental planning. The culture medium was aseptically inoculated with a 24 h inoculum. Initial pH was adjusted to 5.5. Both bioreactors were kept under controlled conditions: aeration rate 1.0 vv^−^^1^m^−^^1^ and 200 rpm mechanical agitation for 120 h at 28°C. At the end of fermentation, samples were collected from the liquid culture to check surface tension and biosurfactant yield.

### Isolation of biosurfactant

Biosurfactant was extracted from the cell-free broth following cell removal by centrifugation at 5000 × g for 20 min and filtered through Whatman no.1 filter paper. Any trace of remaining oil in the metabolic broth was removed before the extraction process. An equal volume of CHCl_3_/CH_3_OH (2:1) was placed with 50 mL of the cell-free broth in a separatory funnel at 28°C. The mixture was vigorously shaken for 15 min and allowed to set until phase separation. The organic phase was removed and the operation was repeated twice. The pooled product from organic phase was dried in an oven until complete evaporation of the solvent at 80°C to a constant weight (Silva et al., 2013).

### Evaluation of the biosurfactant as dispersant

A visual test was used for determination of the dispersant effectiveness of the biosurfactant. Motor oil samples of 5, 10, 20, 40, 80, and 100 μL were carefully added to the surface of seawater (20 mL) contained in a beaker until create 1 cm of deep vortex by slow magnetic stirring. Then 5.0 μL of the cell-free broth or isolated biosurfactant was added to the center of the vortex and stirred to a maximum rate of 2000 rpm over a 1 min period. Deep vortex was created as follows: a magnetic bar was placed inside the beaker containing sea water. The beaker was then placed on a stirring plate, connecting the equipment and increasing stirring of the magnetic bar that when rotating, creates the vortex. The proportions of motor oil added and biosurfactant resulted in biosurfactant/oil ratios of 1/1, 1/2, 1/4, 1/8, 1/16, and 1/20 (v/v). Oil dispersion level was visually estimated after 1 min rest and classified as A, B, C, D, and E to 100% oil dispersion (without any oil surface layer), 75% oil dispersion (with discrete layer on the seawater surface), 50% oil dispersion, 25% oil dispersion and without dispersion, respectively (Sobrinho et al., 2013a).

## Results and discussion

### Optimization of biosurfactant production using RSM

The CCRD matrix and corresponding results are given in Table 2. Multiple regression analysis using RSM was performed to fit the response function to the experimental data and investigate the simultaneous influence of the four variables selected. The best condition for biosurfactant production was found in Run 1 for both response variables, as the lowest surface tension coincided with the highest biosurfactant yield. This occurred using the minimum values of the independent variables *X*_1_, *X*_2_, *X*_3_, and *X*_4_.

**Table 2:** **Experimental deign matrix for optimization of biosurfactant optimization produced by *C. tropicalis* UCP0996 according to CCRD**.

**Runs**	**Sugarcane molasses (%), *X_1_***	**Corn steep liquor (%), *X_2_***	**Waste frying oil (%), *X_3_***	**Inoculum size (%), *X_4_***	**Surface tension (mNm^−1^), *Y_1_***	**Biosurfactant yield (gL^−1^), *Y_2_***
1	−1.0	−1.0	−1.0	−1.0	29.98	4.19
2	−1.0	−1.0	−1.0	1.0	35.07	2.43
3	−1.0	−1.0	1.0	−1.0	32.53	2.90
4	−1.0	−1.0	1.0	1.0	34.20	2.63
5	−1.0	1.0	−1.0	−1.0	30.66	3.21
6	−1.0	1.0	−1.0	1.0	34.28	2.33
7	−1.0	1.0	1.0	−1.0	31.49	3.03
8	−1.0	1.0	1.0	1.0	31.23	3.06
9	1.0	−1.0	−1.0	−1.0	31.76	3.05
10	1.0	−1.0	−1.0	1.0	36.76	1.55
11	1.0	−1.0	1.0	−1.0	34.63	2.44
12	1.0	−1.0	1.0	1.0	35.31	2.31
13	1.0	1.0	−1.0	−1.0	32.35	2.88
14	1.0	1.0	−1.0	1.0	35.87	1.68
15	1.0	1.0	1.0	−1.0	33.04	2.70
16	1.0	1.0	1.0	1.0	32.66	2.95
17	−2.0	0.0	0.0	0.0	32.12	3.13
18	2.0	0.0	0.0	0.0	35.35	2.10
19	0.0	−2.0	0.0	0.0	33.86	2.77
20	0.0	2.0	0.0	0.0	31.96	2.89
21	0.0	0.0	−2.0	0.0	32.95	2.76
22	0.0	0.0	2.0	0.0	32.28	2.90
23	0.0	0.0	0.0	−2.0	31.16	3.18
24	0.0	0.0	0.0	2.0	35.84	1.84
25	0.0	0.0	0.0	0.0	30.10	3.42
26	0.0	0.0	0.0	0.0	30.12	3.44
27	0.0	0.0	0.0	0.0	30.13	3.39
28	0.0	0.0	0.0	0.0	30.14	3.40
29	0.0	0.0	0.0	0.0	30.09	3.38
30	0.0	0.0	0.0	0.0	30.11	3.41

*Mean and standard deviation for replicate experiments at the central point for: Surface tension, 30.12 ± 0.02 mNm^−1^ and Biosurfactant yield, 3.41 ± 0.02 gL^−1^*.

The application of RSM for the estimation of the optimum parameters resulted in an empirical relationship between surface tension and the process variables. The following quadratic polynomial equation best fit the data:

(1) $$\begin{array}{l} Y_{1}=30.115+0.80833X_{1}-0.51917X_{2}-0.12417X_{3}\\ \;+1.17917X_{4}-0.02625X_{1}X_{2}-0.035X_{1}X_{3}-0.08125X_{1}X_{4}\\ \;-0.49X_{2}X_{3}-0.37125X_{2}X_{4}-0.97X_{3}X_{4}+0.91313X_{1}^{\text{2}}\\ \;+0.70688X_{2}^{\text{2}}+0.63313X_{3}^{\text{2}}+0.85438X_{4}^{\text{2}}, \end{array}$$

in which *Y*_1_ is surface tension (mNm^−1^) and *X*_1_, *X*_2_, *X*_3_, and *X*_4_ are coded values for sugarcane molasses, corn steep liquor, waste frying oil and inoculum size, respectively.

ANOVA was performed to validate the quadratic model (Table 3). ANOVA is crucial to testing the significance and acceptability of a model. As can be seen from the table, linear and quadratic terms as well as their interactions were all statistically significant (*p* < 0.05) and the *F*-value (with a 95% confidence interval) was much larger than 4 for each variable and respective interaction. The very low pure error (0.0018) indicated excellent reproducibility of the experimental data. The concentration of the inoculum was the most important factor to the reduction in the surface tension, followed by quadratic term of sugarcane molasses concentration. The correlation between inoculum size and surface tension demonstrates that a minor amount of inoculum is more suitable for reducing surface tension within the experimental limits chosen. Moreover, the correlation coefficient (*R*^2^ = 0.99833) indicates that <1% of the total variation could not be explained by the empirical model. Therefore, the regression model was significant and could adequately be used to describe biosurfactant production.

**Table 3:** **Analysis of variance for response surface quadratic model regarding surface tension achieved with biosurfactant produced by *C. tropicalis* UCP0996^a^**.

**Factor**	**Sum of squares**	**Degrees of freedom**	**Mean square**	**F-ratio**	***p*-value^b^**
*X_1_* (L) ^c^	15.6817	1	15.68167	44804.76	0.000000
*X_1_* (Q) ^d^	22.8699	1	22.86987	65342.48	0.000000
*X_2_* (L)	6.4688	1	6.46882	18482.33	0.000000
*X_2_* (Q)	13.7053	1	13.70530	39157.99	0.000000
*X_3_* (L)	0.3700	1	0.37002	1057.19	0.000001
*X_3_* (Q)	10.9947	1	10.99467	31413.34	0.000000
*X_4_* (L)	33.3704	1	33.37042	95344.05	0.000000
*X_4_* (Q)	20.0217	1	20.02167	57204.77	0.000000
*X_1_* (L) × *X_2_* (L)	0.0110	1	0.01103	31.50	0.002484
*X_1_* (L) × *X_3_* (L)	0.0196	1	0.01960	56.00	0.000673
*X_1_* (L) × *X_4_* (L)	0.1056	1	0.10563	301.79	0.000012
*X_2_* (L) × *X_3_* (L)	3.8416	1	3.84160	10976.00	0.000000
*X_2_* (L) × *X_4_* (L)	2.2052	1	2.20523	6300.64	0.000000
*X_3_* (L) × *X_4_* (L)	15.0544	1	15.05440	43012.57	0.000000
Lack of Fit	0.2074	10	0.02074	59.25	0.000147
Pure Error	0.0018	5	0.00035	–	–
Total square sum	125.2933	29	–	–	–

a *R^2^, 0.99833; adjusted R^2^ = 0.99677*.

b *p ≤ 0.05–significant at 5% level*.

c *(L), linear effect*.

d *(Q), quadratic effect*.

**Figure 1:**
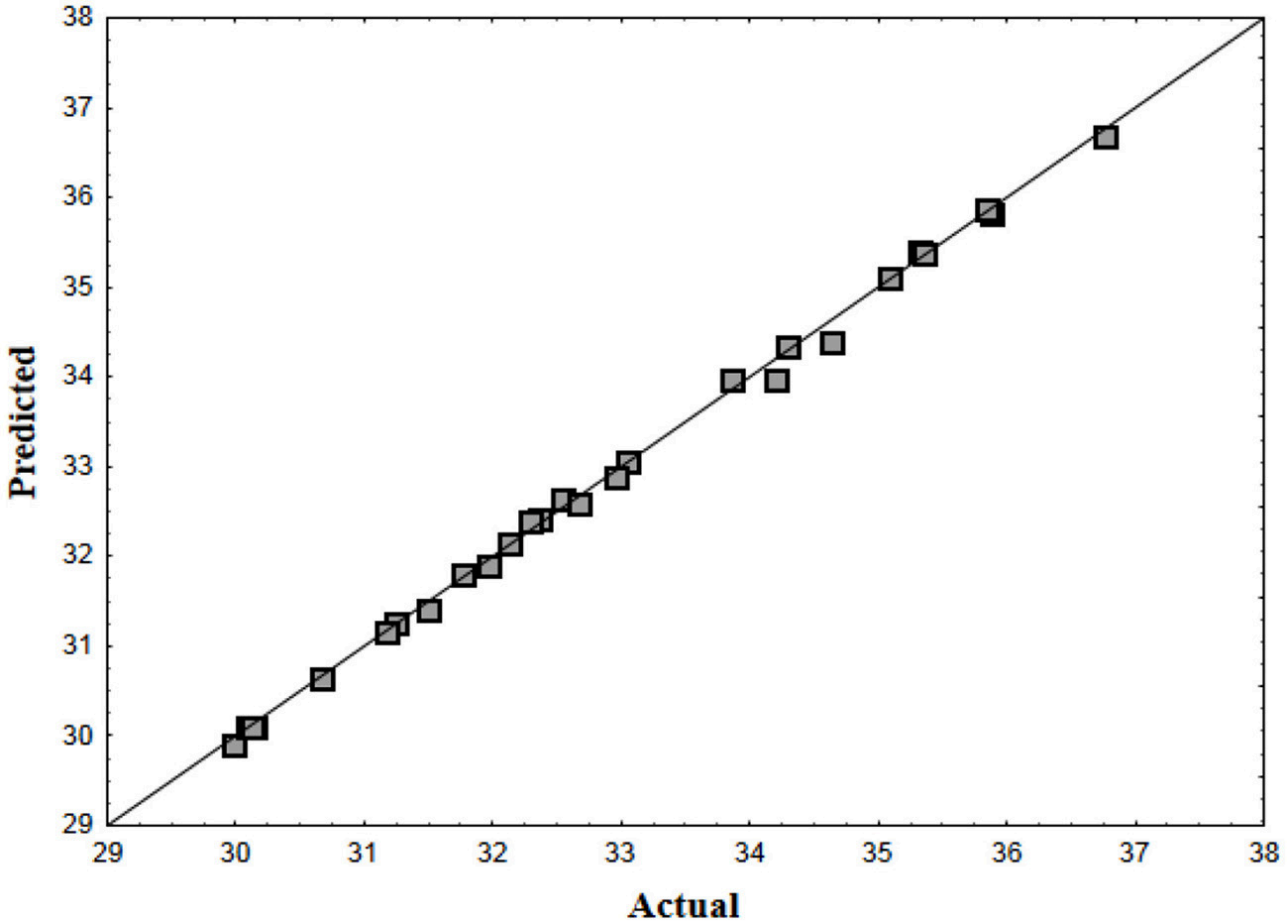
**Plot of predicted vs. actual surface tension achieved using biosurfactant produced by *C. tropicalis* UCP 0996**.

The predicted vs. the actual plot for surface tension determined by the model equation demonstrated that observed values were distributed near the straight line (Figure 1), which indicates that such values were very close to the predicted values (*R*^2^ = 0.99833). Hence, the model proved to be suitable for the prediction of biosurfactant production under the experimental conditions.

**Figure 2:**
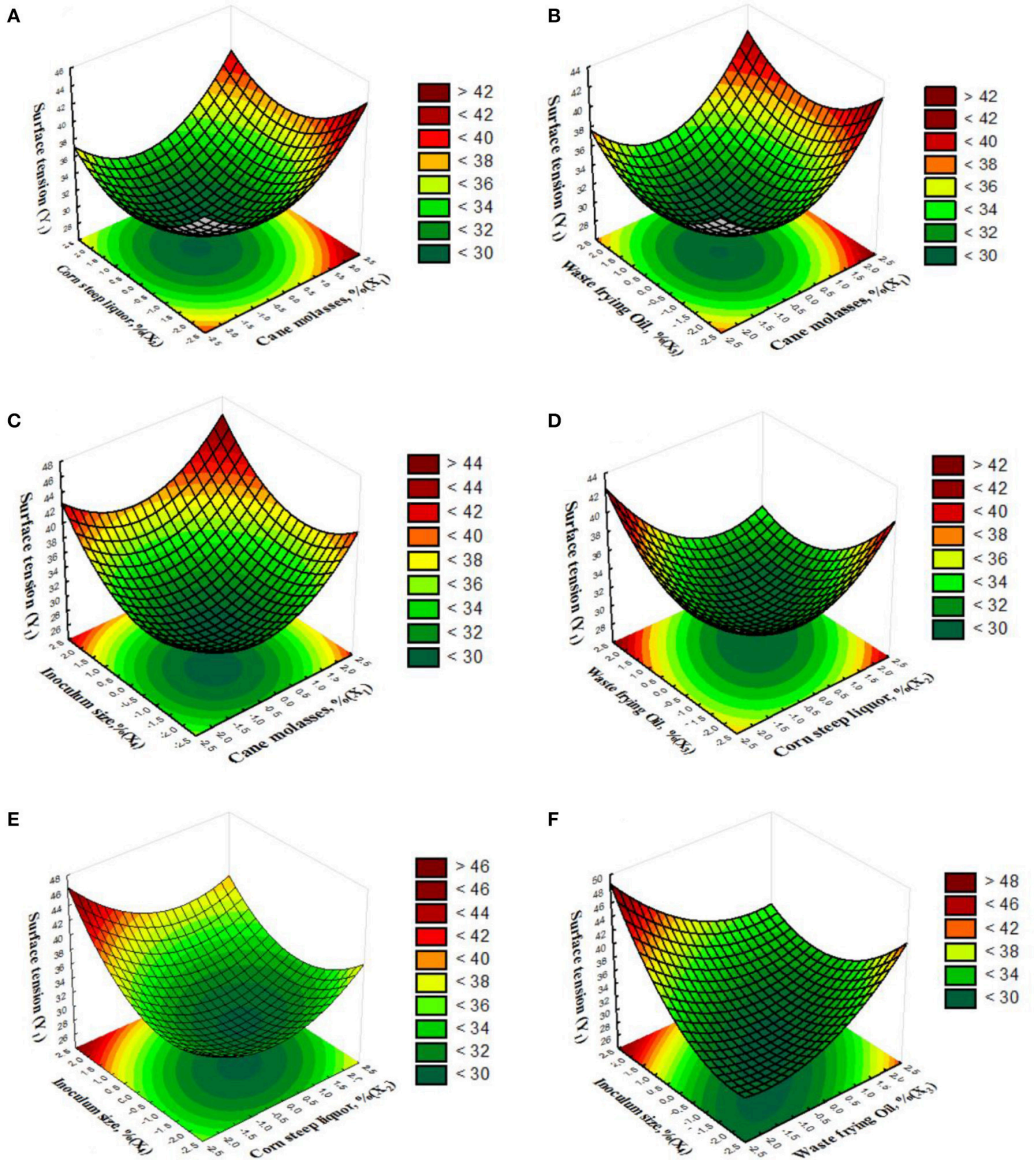
**Response surface plots and contour plots for minimum surface tension generated using data in Table 3**. Inputs, 30 experimental runs carried out under conditions established by CCRD; reduction in surface tension as function of **(A)** sugarcane molasses and corn steep liquor; **(B)** sugarcane molasses and waste frying oil; **(C)** sugarcane molasses and inoculum size; **(D)** corn steep liquor and waste frying oil; **(E)** corn steep liquor and inoculum size; **(F)** waste frying oil and inoculum size.

Figure 2 shows the 3D plots for minimum surface tension (i.e., maximum biosurfactant production) to visualize the interactions of the independent variables, two by two. Figure 2A indicates that a decrease in sugarcane molasses and corn steep liquor near the middle level (with a weak increase from this point) led to a decrease in surface tension. Figure 2B shows that the lowest surface tension was obtained when sugarcane molasses was decreased from the middle level to the minimum level and waste frying oil value was near the middle level. Figure 2C shows that the combination of minimum sugarcane molasses and inoculum size led to a reduction in surface tension. Figure 2D displays a very well-delimited region reflecting the optimized conditions of biosurfactant production with corn steep liquor near the middle level (with a weak increase from this point) and waste frying oil at the middle level. Figure 2E shows that the lowest surface tension was reached when the inoculum size was at the middle level with a weak decreased from this point and corn steep liquor was around the middle level. The combination of minimum inoculum size and waste frying oil concentration (Figure 2F) led to a decrease in surface tension. The elliptical nature of the contour plots presented in Figures 2C–F indicates a high degree of interaction among the factors in each surface response plot analyzed, i.e., it is not possible to predict the biosurfactant properties by modifying only one of the factors studied in each surface response plot. On the other hand, the level curves in Figures 2A,B demonstrate considerable parallelism among the factors and, consequently, a weak interaction, i.e., it is possible to predict surface tension from variations in only of one of the factors in each response surface plot.

Response surface methodology (RSM) was also applied to construct an empirical model for modeling biosurfactant yield and the process variables. The following quadratic polynomial equation best fit the data:

(2) $$\begin{array}{l} Y_{2}=3.406667-0.261667X_{1}+0.024167X_{2}+0.040833X_{3}\\ \;-0.339167X_{4}+0.08625X_{1}X_{2}+0.11125X_{1}X_{3}\\ \;+0.01875X_{1}X_{4}+0.16125X_{2}X_{3}+0.11625X_{2}X_{4}\\ \;+0.32625X_{3}X_{4}-0.195833X_{1}^{\text{2}}-0.142083X_{2}^{\text{2}}\\ \;-0.142083X_{3}^{\text{2}}-0.222083X_{4}^{\text{2}}, \end{array}$$

in which *Y*_2_ is biosurfactant yield (gL^−1^) and *X*_1_, *X*_2_, *X*_3_, and *X*_4_ are coded values for sugarcane molasses, corn steep liquor, waste frying oil and inoculum size, respectively.

**Figure 3:**
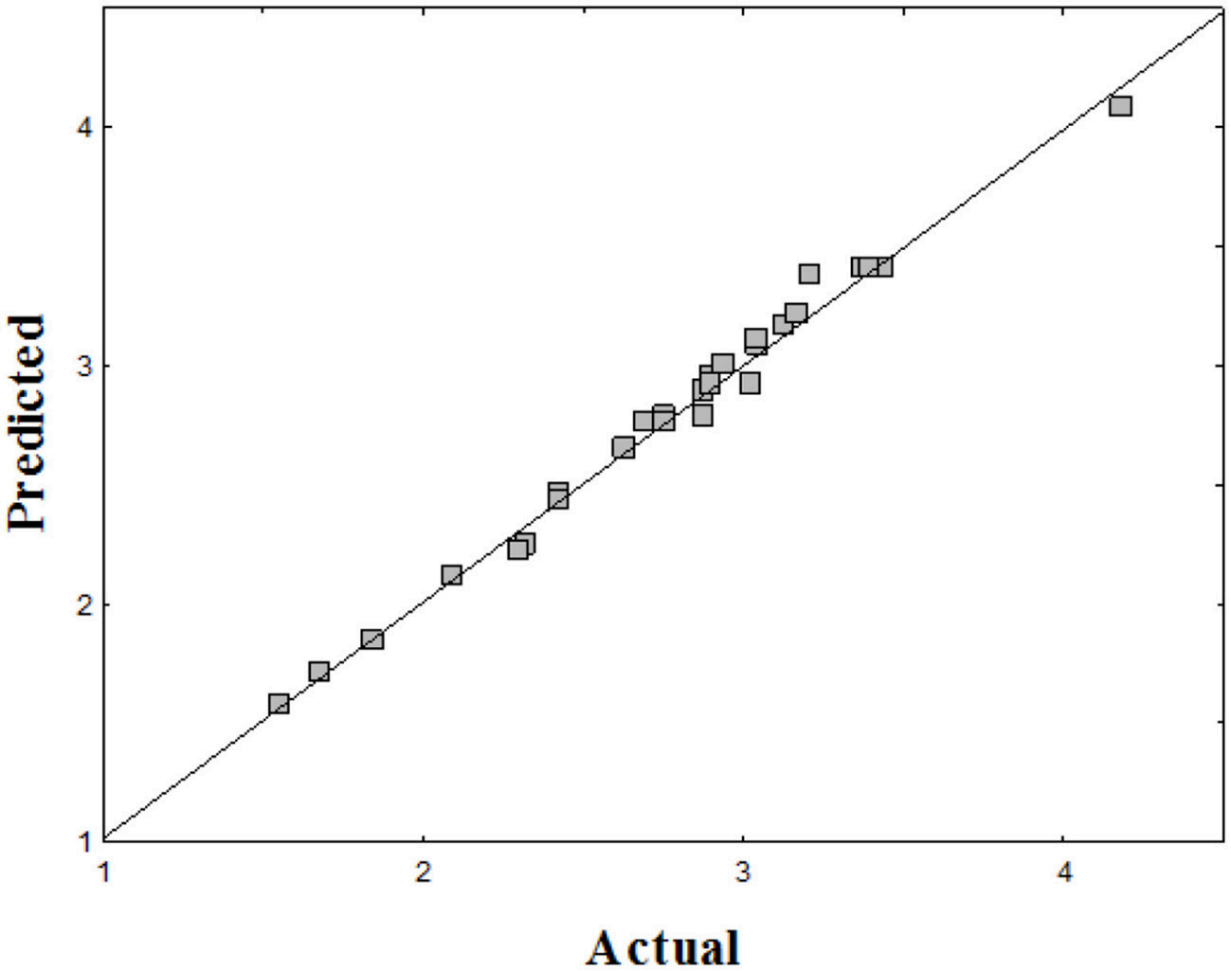
**Predicted vs. actual biosurfactant yield by *C. tropicalis* UCP 0996**.

The evaluation of the empirical model was performed using ANOVA (Table 4). The *p*- and *F*-value (with 95% confidence interval) indicate that all terms were statistically significant (*p* < 0.05; *F* > 4). Reproducibility of the experimental data was once again proven by the relatively low pure error (0.002333). Inoculum concentration was the most important factor to the increase in biosurfactant yield, followed by sugarcane molasses concentration. The explained variance (*R*^2^ = 0.98927) ensured adequate fit (*R* = 0.97925) and predicted vs. actual values were distributed very close to the straight line (Figure 3), thereby validating the forecast model.

**Table 4:** **Analysis of variance (ANOVA) for response surface quadratic model of biosurfactant yield by *C. tropicalis* UCP0996^a^**.

**Factor**	**Sum of squares**	**Degrees of freedom**	**Mean square**	**F-ratio**	***p*-value^b^**
*X_1_* (L)^c^	1.643267	1	1.643267	3521.286	0.000000
*X_1_* (Q)^d^	1.051905	1	1.051905	2254.082	0.000000
*X_2_* (L)	0.014017	1	0.014017	30.036	0.002758
*X_2_* (Q)	0.553719	1	0.553719	1186.541	0.000000
*X_3_* (L)	0.040017	1	0.040017	85.750	0.000247
*X_3_* (Q)	0.553719	1	0.553719	1186.541	0.000000
*X_4_* (L)	2.760817	1	2.760817	5916.036	0.000000
*X_4_* (Q)	1.352805	1	1.352805	2898.867	0.000000
*X_1_* (L) × *X_2_* (L)	0.119025	1	0.119025	255.054	0.000018
*X_1_* (L) × *X_3_* (L)	0.198025	1	0.198025	424.339	0.000005
*X_1_* (L) × *X_4_* (L)	0.005625	1	0.005625	12.054	0.017814
*X_2_* (L) × *X_3_* (L)	0.416025	1	0.416025	891.482	0.000001
*X_2_* (L) × *X_4_* (L)	0.216225	1	0.216225	463.339	0.000004
*X_3_* (L) × *X_4_* (L)	1.703025	1	1.703025	3649.339	0.000000
Lack of Fit	0.102192	10	0.010219	21.898	0.001638
Pure Error	0.002333	5	0.000467	–	–
Total square sum	9.740750	29	–	–	–

a *R^2^, 0.98927; adjusted R^2^, 0.97925*.

b *p ≤ 0.05–significant at 5% level*.

c *(L), linear effect*.

d *(Q), quadratic effect*.

**Figure 4:**
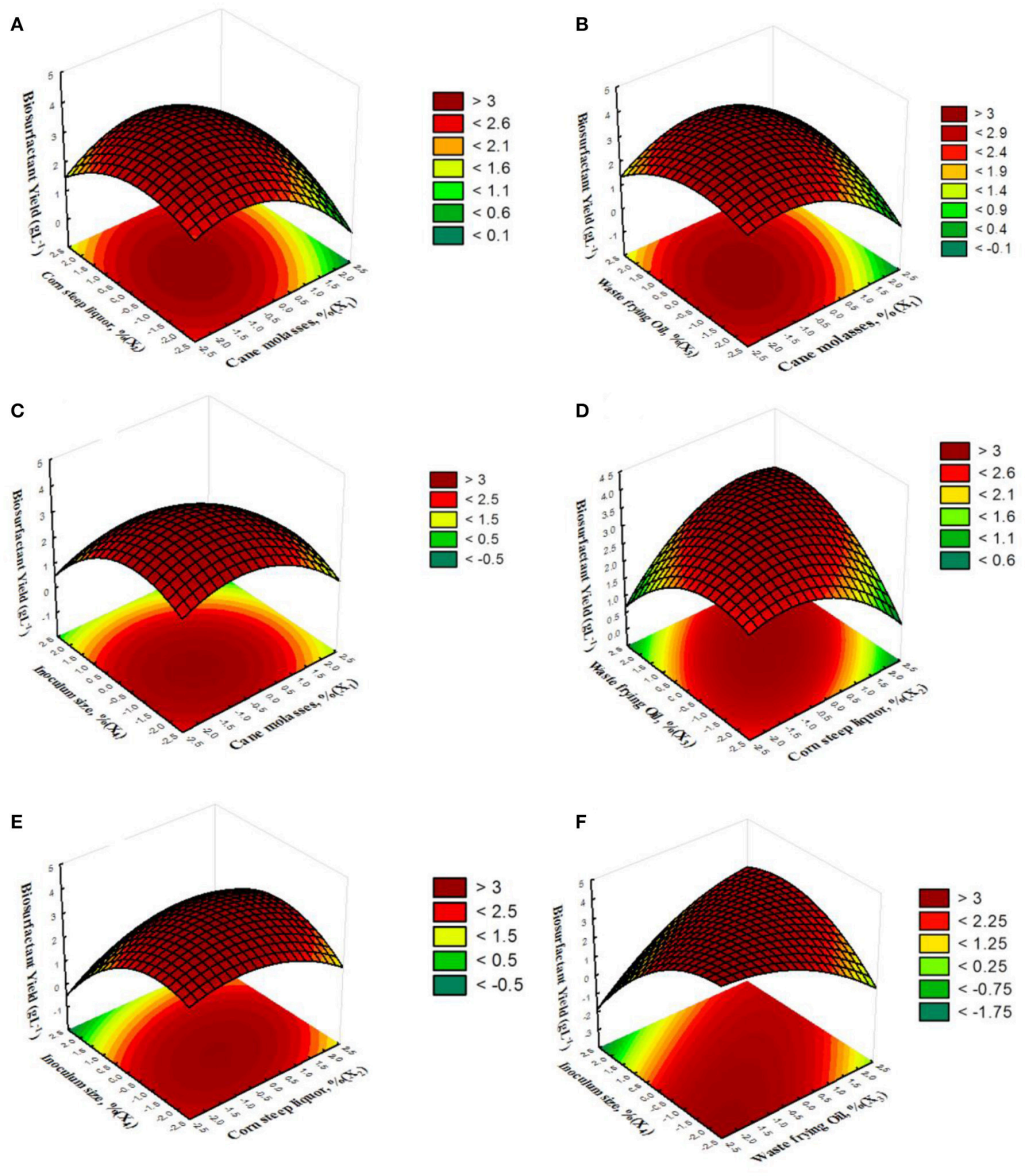
**3D response surface curve of interaction of sugarcane molasses and corn steep liquor (A)**, sugarcane molasses and waste frying oil **(B)**, sugarcane molasses and inoculum size **(C)**, corn steep liquor and waste frying oil **(D)**, corn steep liquor and inoculum size **(E)**, and waste frying oil and inoculum size **(F)** on biosurfactant yield.

Figure 4 displays the three-dimensional plots for biosurfactant yield. The interactions between the variables shown in Figures 4A–F are very similar to those found in Figures 2A–F for surface tension. Biosurfactant yield increased when sugarcane molasses decreased and corn steep liquor remained near the middle level (Figure 4A). The curves in this figure demonstrate considerable parallelism between the factors and, consequently, a weak interaction. A similar effect was found in the relationship between sugarcane molasses and waste frying oil (Figure 4B). The combination of minimum sugarcane molasses concentration and inoculum size (Figure 4C) led to an increase in biosurfactant yield. The curves indicate a reasonable interaction. Around middle level corn steep liquor and inoculum size with a weak decreased from this point produced satisfactory biosurfactant yield, with a strong interaction between the variables (Figure 4D). Figure 4E shows that biosurfactant yield increased when corn steep liquor and inoculum size decreased tending toward the minimum levels. The elliptical nature of the contour plots indicates a high degree of interaction between the factors. Figure 4F shows that the combination of minimum inoculum size and minimum waste frying oil led to the best biosurfactant yield under the conditions tested (local maximum in the region studied). The very sinuous curves indicate the high degree of interaction between these factors. It is important to consider that higher substrate concentrations did not necessarily stimulated the biosurfactant production. This can be explained by the fact that when the substrate is present in high concentrations it can be directed to the production of cellular biomass. RSM demonstrated that the interaction between the three substrates (sugarcane molasses, waste frying oil and corn steep liquor) should be considered simultaneously, rather than individually. Regarding the inoculum size, the results showed that the minimum inoculum size favored the production of the biosurfactant. The higher number of cells may increase the competition for the nutrients for cell maintenance rather than the production of biosurfactant (Ghribi and Ellouze-Chaabouni, 2011; Onwosi and Odibo, 2013; Nalini and Parthasarathi, 2014). Inoculum size has demonstrated high influence on biosurfactant production in previous studies and can have a definite effect on economics of a microbial process (Mnif et al., 2013; Onwosi and Odibo, 2013). The CCRD conducted by Lima and Souza (2014) showed that the inoculum concentration was the factor that had the greatest effect on biosurfactant production by *Bacillus subtilis* PC, providing the best surface tension values of the 32 mNm^−1^. In another study, Ghribi and Ellouze-Chaabouni (2011) studied the effect of inoculum size on biosurfactants production by *B. subtilis* SPB1 where adequate inoculum size reached lipopeptide biosurfactants production of 2.04 gL^−1^. Kiran et al. (2010) also found that a new glycolipid biosurfactant from marine *Nocardiopsis lucentensis* MSA04 was critically controlled by inoculum size by either independently and/or interactively with others studied variables.

Few studies have been conducted using sugarcane molasses as the carbon source in the cultivation of microorganisms for biosurfactant production. In a study conducted by Santos et al. (2010), biosurfactants were synthesized by *Pseudomonas aeruginosa* (P.A.) using sugarcane molasses as carbon and energy source in a concentration of 3.0%. The results showed a maximum rhamnolipid production of 4.47 gL^−1^. Daverey and Pakshirajan (2009) obtained minimum surface tension of 34.15 mNm^−1^ by production of sophorolipid when cultivated the yeast *C. bombicola* in a cheap fermentative medium containing sugarcane molasses. Joshi et al. (2008) also tested the biosurfactant production using sugarcane molasses for cultivation of *Bacillus licheniformis* K51, *B. subtilis* 20B, *B. subtilis* R1, and *Bacillus* strain HS3, which had surface tension values of 29.67, 29.33, 30.33, and 30.67 mNm^−1^, respectively.

On the other hand, the use of corn steep liquor as the nitrogen source for biosurfactant produced by microorganisms has been widely studied (Luna et al., 2013; Silva et al., 2013, 2014a; Campos et al., 2014; Santos et al., 2014). In a study by Luna et al. (2013), the utilization of corn steep liquor as low cost constituent to *C. sphaerica* growth resulted in reducing the surface tension of cell-free broth to 25 mNm^−1^ and biosurfactant yield was 9 gL^−1^. Corn steep liquor also was used by Santos et al. (2013) as a constituent of low cost for the cultivation of *C. lipolytica* UCP0988, obtaining a reduction in surface tension from 50 to 28 mNm^−1^. Rocha e Silva et al. (2014) used 2.0% corn steep liquor as nitrogen source on *Pseudomonas cepacia* medium growth and as a result, it was obtained surface tension of 27.57 mNm^−1^ at the end of the cultivation e yield of 5.2 gL^−1^ of isolated biosurfactant.

Microorganisms have been also studied using waste frying oil as the carbon source. Batista et al. (2010) report a maximum biosurfactant yield of 3.61 gL^−1^ by *C. tropicalis* using waste frying oil as substrate, with a 33.66 mNm^−1^ reduction in surface tension. Oliveira and Garcia-Cruz (2013) tested different concentrations waste frying oil as alternative carbon sources on *Bacillus pumilus* cultivation, which obtained surface tension reduction of 45 mNm^−1^ and maximum crude biosurfactant production of 5.7 gL^−1^ for waste frying oil, in concentration of 5%. Using waste frying oil as substrate for biosurfactant production by *C. tropicalis* CECT 1440, Haba et al. (2000) found a reduction of 35 mNm^−1^ in mean surface tension.

**Figure 5:**
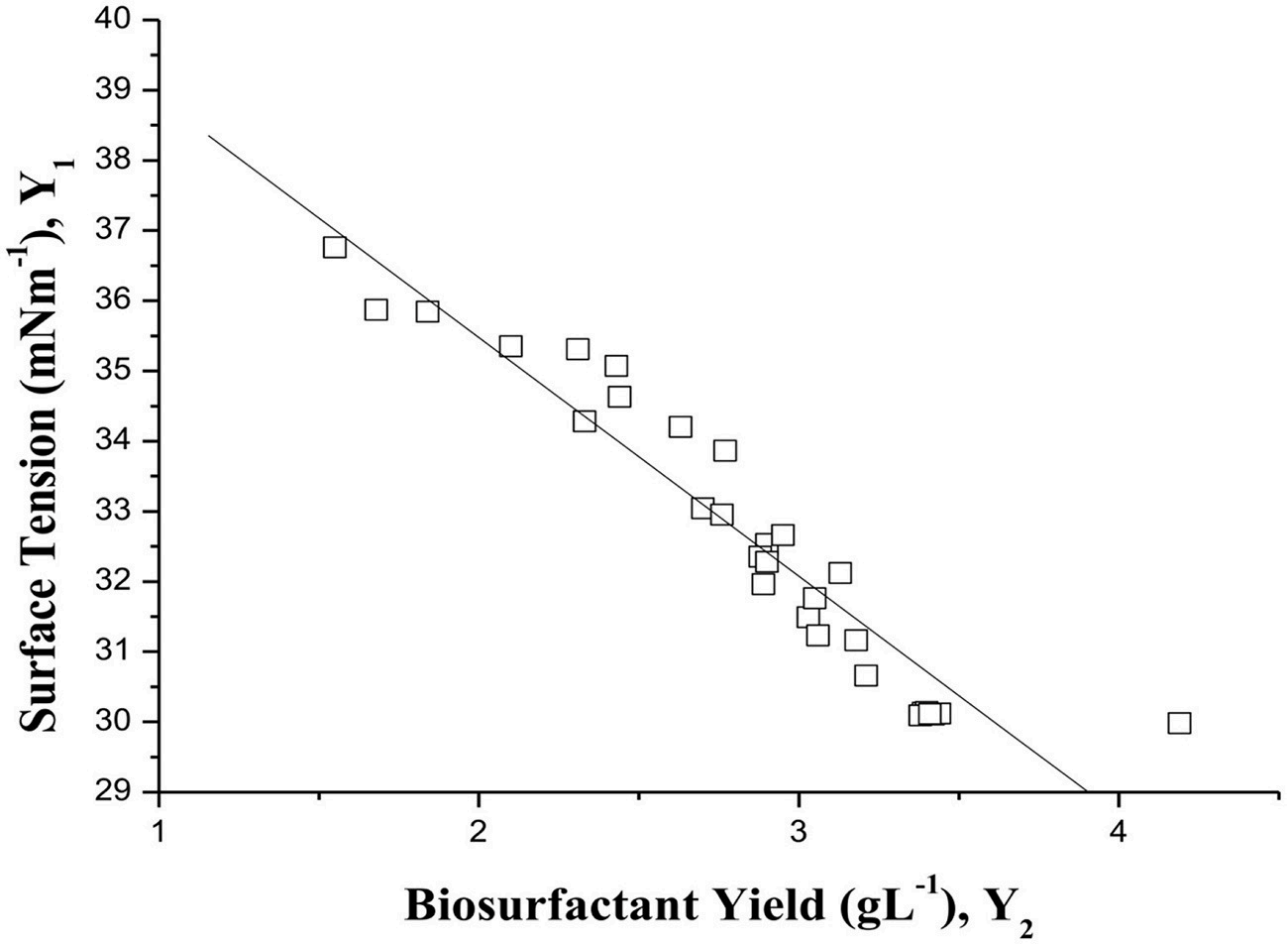
**Comparative parity plot of surface tension (*Y*_1_) and biosurfactant yield (*Y*_2_) based on correlation coefficients**.

A statistical correlation test was used to determine the association between surface tension (*Y*_1_) and biosurfactant yield (*Y*_2_) in the CCRD (Table 2). A strong negative correlation was found between *Y*_1_ and *Y*_2_ (Pearson's bivariate correlation coefficient: *r* = −0.95, *p* < 0.0001) (Figure 5), i.e., an increase in biosurfactant concentration resulted in a decrease in surface tension in all experiments performed. Determination coefficient (R^2^) for both models (Tables 3, 4) was very close to 1, which means that either model can be applied to explain the variability between experimental and predicted values in its respective responses (*Y*_1_ or *Y*_2_). The very low coefficient of variation (CV) for surface tension (CV = 6.38%) and reasonably low CV for biosurfactant yield (CV = 20.37%) indicate a high degree of precision and adequate reliability of the experimental values for both *Y*_1_ and *Y*_2_, although the CV for *Y*_2_ was much smaller. The use of statistical models to enhance the fermentation process has been increasing in current biotechnology, due to the applicability and suitability of this approach (Zhu et al., 2013).

As the high production cost is the major drawback regarding biosurfactants, economical processes that employ low-cost materials at the key to successful production (Rufino et al., 2014; Silva et al., 2014b). Analyzing the effectiveness of the process with respect to production costs, the conditions established in Run 1 of the present study proved to be the most suitable, since the constituents of the medium (sugarcane molasses, corn steep liquor, waste frying oil and inoculum) were used at their lowest concentrations (−1). Therefore, the use of industrial byproducts proved to be a promising way to reduce biosurfactant production costs.

Previous studies have already demonstrated the efficiency of the dual application of industrial wastes and RSM in enhancing of economic production of biosurfactant by microorganisms. High yields in biosurfactants have been reached through the combination between a vegetable oil and carbohydrate as substrate (Santos et al., 2016). Regarding the use of waste frying vegetal oils, isolated or combined with a soluble substrate, promising results have been obtained in the last decades for *Candida* species in our laboratories (Sarubbo et al., 1999, 2001, 2006, 2007; Coimbra et al., 2009; Campos et al., 2014). Thus, waste frying oil and molasses were used as the insoluble and soluble carbon sources, respectively, while corn steep liquor was used as the nitrogen source. Luna et al. (2011b) reported the utilization of a statistical experimental design and RSM to optimize the concentrations of two agro-industrial residues, soybean oil and corn steep liquor, for biosurfactant production by *C. sphaerica* UCP0995, reaching a minimum surface tension of 25.25 mNm^−1^. Similarly, Silva et al. (2013) also reported the utilization of a CCRD and RSM to biosurfactant production from a new strain *Pseudomonas cepacia* CCT6659 cultivated in a low cost medium formulated with 2% waste frying oil, 3% corn steep liquor and 0.2% NaNO_3_ allowed to achieve a maximal reduction in surface tension of 26 mNm^−1^ and biosurfactant yield of 8.0 gL^−1^. In another study conducted by Mnif et al. (2012), a central composite design and RSM were employed to increase the yield of a lipopeptide biosurfactant produced by *B. subtilis* SPB1 using also low-cost substrates, where was obtained biosurfactant yield about 4.5 gL^−1^ with an optimal medium composed of sesame peel flour (33 gL^−1^) and diluted tuna fish cooking residue (40%).

Based on the results of the CCRD (ANOVA, Tables 3, 4), the condition performed with 2.5% sugarcane molasses, 2.5% corn steep liquor, 2.5% waste frying oil, and 2% inoculum size (Run 1) was selected for biosurfactant production. Among the variables studied, the most significant in both models were inoculum size and sugarcane molasses, respectively.

### Scale up of biosurfactant production

To confirm the efficiency of the statistically optimized variables, experiments in bench-scale and pilot bioreactors were carried out using the optimum concentrations of the variables in Run 1. Table 5 displays the results of the biosurfactant produced in bioreactors. As can be seen, the yield of the biosurfactant produced by *C. tropicalis* had an increase of 40 and 75% in values when produced in 2- and 50-L bioreactors, respectively, compared to the best results found in flasks fermentation (Run 1). This excellent result is probably due to the better control of aeration, agitation and temperature, once bioreactors are systems completely closed, favoring cell growth and a greater biosurfactant yield. In addition, the cultivation conducted in shaker uses orbital shaking while the bioreactor uses mechanical agitation, where the oxygen supply is continuous, allowing better yields of the biosurfactant (Luna et al., 2015). In a similar study performed by Sobrinho et al. (2013b) *C. sphaerica* UCP 0995 grown in distilled water supplemented with 5.0% soybean oil refinery residue and 2.5% corn steep liquor presented a biosurfactant yield of 6.36 gL^−1^ after 144 h of culture in a 5-L bioreactor, whereas with the same culture medium Sobrinho et al. (2008) reported a yield of 4.5 gL^−1^ after 144 h of a culture of *C. sphaerica* UCP 0995 using flasks.

**Table 5:** **Surface tension and biosurfactant yield evaluation of the biosurfactant from *C. tropicalis* UCP0996 grown in distillated water supplemented with 2.5% sugarcane molasses, 2.5% waste frying oil, and 2.5% corn steep liquor in bioreactors**.

**Vessel type**	**Biosurfactant yield (gl^−1^)**	**Surface tension (mnm^−1^)**
2-L bioreactor	5.87 ± 0.21	34.12 ± 0.07
50-L bioreactor	7.36 ± 0.34	35.6 ± 0.05

*Results are expressed as means ± standard deviations of values obtained from triplicate experiments*.

Biosurfactant produced by *C. tropicalis* decreased the surface tension of water from 72 to a minimum of 29. 98 mNm^−1^ when produced in shaker (Run 1). However, it had a remarkable variation in the surface tension when produced in bioreactors (Table 5). This same phenomenon was also observed on a recent study carried out by Luna et al. (2015) for *C. sphaerica* cultivated in a medium with 9.0% of refinery residue of soybean oil and 9% of corn steep liquor during 144 h. Shake-flask method reported a surface tension of 25 mNm^−1^ while in the bioreactor the surface tension was 27.48 mNm^−1^. This may be related to the intrinsic effects of the scaling up, since the increase in volume promotes a consequent increase in the total surface area for complete saturation with biosurfactants, which explains this small variation observed in surface tension. When the issue is the production in large scale of biosurfactants, the use of bioreactors becomes the most promising alternative that will make the microbial production more favorable from the technical and economic points of view, compared to the limitations in relation to benchtop techniques (Marti et al., 2014; Luna et al., 2015).

### Evaluation of the biosurfactant as dispersant

Many processes of the oil industry are carried out in the marine environment. Eventually, a part of the process oil reaches accidentally the seawater and, in turn, surfactants must be used in conjunction with other containment measures. Dispersion is a process by which a hydrocarbon is dispersed into the aqueous phase as very small emulsions. Dispersion is related to both the interfacial tension and surfactant concentration, and differs from displacement in that displacement process is only related to the interfacial tension between aqueous and hydrophobic phases and no emulsion formation (Almeida et al., 2016). In this study, the biosurfactant from *C. tropicalis* UCP0996 was tested as an oil dispersant in seawater. As a result, the maximum motor oil dispersion achieved by the biosurfactant produced under optimized conditions at a biosurfactant/oil ratio of 1/1 (v/v) was 50 and 75% for the cell-free broth and isolated biosurfactant, respectively (Table 6). Similar results were obtained by Sobrinho et al. (2013a) for the biosurfactant produced by *C. sphaerica* cultivated in 5% vegetal oil refinery waste and 2.5% corn steep liquor that showed an oil spreading efficiency of 75%.

**Table 6:** **Evaluation of the biosurfactant from *C.tropicalis* UCP0996 grown in distillated water supplemented with 2.5% sugarcane molasses, 2.5% waste frying oil, and 2.5% corn steep liquor as an oil dispersant**.

**Cell-free broth**		**Isolated biosurfactant**	
**Biosurfactant/oil ratio**	**Dispersion classification**	**Biosurfactant/oil ratio**	**Dispersion classification**
1/1	C (50%)	1/1	B (75%)
1/2	C (50%)	1/2	C (50%)
1/4	D (25%)	1/4	D (50%)
1/8	D (25%)	1/8	D (25%)
1/16	D (25%)	1/16	D (25%)
1/20	E	1/2	E

## Conclusion

The present study demonstrated the effectiveness of using a central composite rotational design to identify the optimum culture conditions for the production of biosurfactant from *C. tropicalis* UCP0996. The feasibility of the application of bioreactors and its combination with the use of industrial and agricultural wastes proved to be important tools toward a high yield and low cost for biosurfactant production, especially if the process is going to be implemented on an industrial scale and commercial application of this promising biosurfactant. The biomolecule exhibited considerable potential regarding the dispersion of oil from water surface demonstrating its potential application in the oil industry.

## Author contributions

All authors contributed in this work. DA carried out the experiments. VS analyzed the data. LS designed the project. DA, RS, JL, RR, and LS wrote the manuscript. LS performed manuscript editing and final improvement.

## Funding

This study received funding from the Brazilian fostering agencies the State of Pernambuco Foundation for the Assistance to Science and Technology (FACEPE); the Research and Development Program of the Brazilian National Electrical Energy Agency (ANEEL); the National Council for Scientific and Technological Development (CNPq), and the Federal Agency for the Support and Evaluation of Graduate Education (CAPES).

### Conflict of interest statement

The authors declare that the research was conducted in the absence of any commercial or financial relationships that could be construed as a potential conflict of interest.
